# Intestinal brush border assembly during the peri-hatch period and its contribution to surface area expansion

**DOI:** 10.1016/j.psj.2021.101401

**Published:** 2021-07-28

**Authors:** Naama Reicher, Zehava Uni

**Affiliations:** Department of Animal Science, The Robert H. Smith, Faculty of Agriculture, Food and Environment, The Hebrew University of Jerusalem, Rehovot 76100, Israel

**Keywords:** enterocytes, perihatch, brush-border, microvilli, gene expression

## Abstract

Microvilli generate the small intestinal brush border, the main site of nutrient digestion and absorption. Mucosal structuring of the small intestine of chicken during the perihatch period has been widely researched, yet the developmental dynamics of microvilli during this period have not been fully characterized. In this study, we examined the structural and molecular characteristics of microvilli assembly and maturation during the perihatch period. Small intestines of broiler embryos and chicks were sampled at prehatch ages 17 E and 19 E, at day of hatch (**DOH**) and at 1, 3, 7, and 10 d posthatch. Morphological evaluations and measurements were conducted by scanning electron microscopy (**SEM**) and light microscopy (**LM**) (n = 3/timepoint), and expression of microvilli structural genes Plastin 1, Ezrin, and Myo1a was examined by Real-Time qPCR (n = 6/timepoint). Results revealed dissimilar patterns of microvilli and villi development during the perihatch period. From 19 E to 1 d, microvilli lengths increased 4.3-fold while villi lengths increased 2.8-fold (*P* < 0.0001). From 3 to 7 d, villi lengths increased by 20% (*P* < 0.005), while microvilli lengths decreased by 41% (*P* = 0.001). At 10 d, microvilli lengths stabilized, while villi continued to elongate by 26% (*P* < 0.0001). Estimations of the microvilli amplification factor (**MAF**) and total enterocyte surface area (**TESA**) revealed similar trends, with peak values of 78.53 and 1961.67 µm^2^, respectively, at 3 d. Microvilli structural gene expression portrayed diverse patterns. Expression of Plastin 1, which bundles and binds actin cores to the terminal web, increased 8.7-fold between 17 E and DOH (*P* = 0.005), and gradually increased up to 7 d (*P* = 0.045). Ezrin and Myo1a, both actin core-cell membrane cross-linkers, portrayed different expression patterns throughout the perihatch period, as Ezrin expression was relatively stable, while Myo1a expression increased 15.8-fold between 17 E and 10 d (*P* < 0.0001). We conclude that microvilli assembly during the perihatch period is a rapid, coordinated process, which dramatically expands the digestive and absorptive surface area of the small intestine before the completion of villi maturation.

## INTRODUCTION

The developmental dynamics of the small intestine during the perihatch period have been widely researched, with emphasis on mucosal morphometric expansion, as well as expression and activity of nutrient transporters and digestive enzymes (Uni et al., 1995, 1998, 2003; Geyra et al., 2001a; Iji et al., 2001; Gilbert et al., 2007, 2010; de Oliveira et al., 2009; Zwarycz and Wong, 2013). These developmental processes govern the transition from embryonic, egg-based nutrition to posthatch exogenous feeding (Noy and Sklan, 1998; Moran, 2007) and are vastly affected by initial posthatch feeding (Noy and Sklan, 1999; Geyra et al., 2001b; Uni and Ferket, 2004; Reicher et al., 2020) as well as embryonic, in-ovo nutrition (Uni and Ferket, 2004; Tako et al., 2004; Foye et al., 2007; Cheled-Shoval et al., 2011). Studies have regularly focused on villi lengths, crypt depths and the villus/crypt ratio as indicative parameters of the developmental and functional status of the perihatch small intestine. However, the developmental dynamics of microvilli, which are key factors for generating the functional, surface-amplifying brush border of the small intestine, have not been fully characterized during the perihatch period.

Microvilli are tightly packed, finger-like projections from enterocyte apical surfaces, which constitute the primary cellular interface with luminal contents in vertebrates. Microvilli membranes house nutrient transporters and channels as well as digestive enzymes (McConnell et al., 2011), some of which are packaged into vesicles and secreted into the intestinal lumen from microvilli tips (McConnell et al., 2009).

Microvilli biostructuring initiates at enterocyte apical membranes during differentiation by actin core helical bundling and anchoring into an underlying terminal web, as previously characterized in mammalian cells (Mooseker and Tilney, 1975; Ohta et al., 2012; Crawley et al., 2014). The exceptionally uniform size, orientation, and density of microvilli are generated by a complex network of proteins and signaling molecules, such as Villin and Plastin 1, which bundle actin filaments, in coordination with Ezrin and Myosin 1a (**Myo1a**), which crosslink the core actin bundles to their surrounding membrane (reviewed by Crawley et al., 2014).

The contribution of microvilli to the amplification of the intestinal mucosal apical surface area is several magnitudes higher than villi surface area amplification in adult humans (Helander and Fändriks, 2014), mice (Ferraris et al., 1989), and chicken (Mitjans et al., 1997). This indicates the fundamental significance of microvilli in generating sufficient digestive and absorptive capacities of the small intestine within its limited volume and length.

In chicken, transmission electron microscopy (**TEM**) revealed that the formation of microvilli initiates during the 9th d of incubation (9 E). Microvilli gain uniform internal structuring by 11 E and develop rootlets which straighten their orientation by 15 E. Their lengths measure approximately 0.5 µm between 9 E and 19 E, and increase significantly during the last 2 d of embryonic development and the first 5 d posthatch (Chambers and Grey, 1979). Surface area amplification by microvilli in chicken has been calculated at day of hatch (**DOH**) and several weeks posthatch (Ferrer et al., 1995; Mitjans et al., 1997). However, structural and molecular development of microvilli and their contribution to surface area amplification during the perihatch period has not yet been evaluated.

In this study, we visualized ultrastructural changes within the apical surface of the small intestine of chick embryos at 17 E and 19 E and hatchlings from DOH and up to 10 d, by scanning electron microscopy (**SEM**). During these perihatch timepoints, we compared microvilli and villi morphometric parameters, estimated microvilli amplification factors and total enterocyte surface areas, and quantified relative expression of microvilli structural genes Plastin 1, Ezrin, and Myo1a by Real-Time qPCR.

## MATERIALS AND METHODS

### Experimental Animals and Sample Collection

All procedures were conducted according to established guidelines for animal care and handling and approved by the Hebrew University Institutional Animal Care and Use Committee (IACUC:AG-17-15355-2). Fertile Cobb500 broiler eggs (n = 75) of equal weights (64 g ± 2.7 SD) were obtained from a commercial hatchery (Brown Ltd., Hod-Hasharon, Israel) at day of lay. Eggs were immediately incubated in a Petersime 9600 incubator (Petersime, Zulte, Belgium) at the Faculty of Agriculture of the Hebrew University under standard conditions (37.8°C, 60% relative humidity). Embryo sampling was conducted at embryonic days 17 and 19 (17 E and 19 E, respectively). At both timepoints, 6 eggs were randomly selected for sampling. Sampled embryos were euthanized by cervical dislocation. Their small intestines were immediately collected and separated at the midj-ejunum segment (midway between the duodenal loop and yolk stalk). At each sampling timepoint, jejunum samples from all 6 embryos were processed for RNA extraction. A second jejunum sample was separated from 3 of the embryos (randomly selected) and processed for histological and scanning electron microscopy procedures. Hatching window was monitored between 20 E and 21 E, and chicks hatched within a 6 h range (n = 30) were housed in a single brooder at the Faculty of Agriculture of the Hebrew University. Chicks were immediately granted ad libitum access to water and starter feed (nutrient composition is detailed in Table 1). Chick sampling was conducted at DOH (prior to housing), and d 1, 3, 7, and 10. No chick mortalities were recorded during the experiment. At each sampling timepoint, 6 chicks were weighed and euthanized by cervical dislocation. Mid-jejunum samples were collected and divided for RNA extraction (n = 6 at each timepoint) and histological and scanning electron microscopy procedures (n = 3 at each timepoint) as described for embryo sampling. Average BW ± SD for all chicks/embryos, followed by average BW ± SD values for SEM and histological procedures were as follows: DOH: 41.5 ± 2.8 g; 41.4 ± 0.3 g. 1 d: 46.8 ± 2.9 g; 46.23 ± 0.4 g. 3 d: 83.6 ± 4.9 g; 83.9 ± 2.8 g. 7 d: 191.6 ± 21.8 g; 198.4 ± 13.3 g. 10 d: 330.1 ± 26.4 g; 335.5 ± 21.24 g.

**Table 1:** Nutritional composition of starter feed.

Item	Amount
Protein %	22.5
Calcium %	1
Total phosphorus %	0.75
Av. Phosphorus %	0.45
Total fat %	5
Total fiber %	3.5
Ash %	5.5
NaCl%	0.33
Linoleic acid %	2
Moisture %	12
Mn (g)	100
Vit A (MIU)	12
Vit D (MIU)	5
Vit E (IU)	100,000
Met. En. (Kcal/Kg)	3030

### Histological Procedures

At each sampling timepoint, a 1 cm jejunum sample was separated from three birds. Samples were rinsed in PBS and fixed in 3.7% formaldehyde in PBS (pH 7.4) for 24 h at room temperature (RT). Tissues was then rinsed in PBS and dehydrated in graded series of ethanol, cleared by Histochoice (Sigma-Aldrich, Rehovot, Israel) and embedded in Paraplast (Sigma-Aldrich). Tissue blocks were sectioned 5 µm thick with a microtome, and mounted on SuperFrost Plus glass slides (Bar-Naor Ltd., Petah-Tikva, Israel) and stained with hematoxylin and eosin (Sigma-Aldrich). Sections were visualized at ×200 magnification by a BX40 Olympus microscope (Waltham, MA), and images were captured using cellSense Imaging Software and analyzed using FIJI software. Villi lengths were measured from base to tip in 10 intact villi from each bird at each timepoint.

### Scanning Electron Microscopy

At each sampling timepoint, a 0.5 cm jejunum sample was separated from 3 birds. Samples were rinsed in phosphate buffer (pH 7.4) and fixed in 2% glutaraldehyde and 4% formaldehyde in 0.2M CaCo buffer, pH 7.4 (Sigma-Aldrich) for 24 h at RT. Tissues were then washed 3 times with 0.2M CaCo buffer, followed by postfixation in 0.1% OsO4 (Sigma-Aldrich) in 0.2M CaCo buffer for 1 h, three washes with phosphate 0.2M CaCo buffer and four washes in double distilled water. Tissues were then dehydrated in a graded series of 20% to 100% ethanol, and critical point dried (K850 Critical Point Dryer, Quorom Technologies Ltd., East Sussex, UK). Samples were then mounted on aluminum stubs with carbon tape and villi tips were trimmed off using a razor blade under a stereomicroscope at several locations within each sample for visualizing microvilli lengths. Samples were sputter-coated with iridium (Q150T ES Quorom Technologies Pvt. Ltd.) and visualized by a JEOL 7800F high-resolution scanning electron microscope (Jeol Ltd., Tokyo, Japan). Images were captured at 3kv and 4 WD and analyzed using FIJI software. Microvilli lengths and diameters were measured at X12,000 magnified side-views of enterocyte apical membranes in 6 cells from 3 villi per replicate (n = 3 embryos/chicks at each timepoint). Microvilli densities were measured in 3 to 5 perpendicularly oriented cells with prominent borders at ×12,000 magnification, from 3 villi per replicate (n = 3 embryos/chicks at each timepoint). Enterocyte surface areas were measured in 5 perpendicularly oriented cells with prominent borders at ×6,000 magnification from 3 villi per replicate (n = 3 embryos/chicks at each timepoint).

### Real-Time Quantitative PCR

At each sampling timepoint, a 500 mg jejunum sample was separated from 6 embryos/chicks. Samples were flash frozen in liquid nitrogen upon collection. Total RNA was extracted using Trizol reagent (Sigma-Aldrich) according to the manufacturer's protocol. cDNA was synthesized from 1.0 μg total RNA using a PCRBIO 1-Step Go RT-PCR Kit (Tamar, Mevaseret Zion, Israel). Primers for Plastin 1, Ezrin, and Myo1a were designed using Primer Blast (https://www.ncbi.nlm.nih.gov/tools/primer-blast/) to span exon-exon junctions, and validated for exclusion of genomic DNA contamination on pooled cDNA and gDNA samples by 1.5% agarose gel electrophoresis. Primer sequences are listed in Table 2.

**Table 2:** Primer list for real-time PCR.

Gene Type	Gene name	Accession number	Forward primer (5′)	Reverse primer (3′)	Product length
Target	Plastin 1	NM_205347.1	GCAGCGGT-GGGGAGTAT	ACCCACTGTTGT-CAATATCTATCT	139
Target	Ezrin	NM_204885.1	GTGAAGGAAGG-GATCCTCAGTG	TGCTGATCCAT-CACCCTCTGG	167
Target	Myo1a	NM_205163.1	GATGCGCAAG-AGCCAAATCC	GGTACATCCTG-CGGGTCTTC	134
Reference	β-actin	NM_205518.1	AATGGCTCCG-GTATGTGCAA	GGCCCATACCA-ACCATCACA	112

Quantification of Plastin 1, Ezrin and Myo1a mRNA expression by Real-Time qPCR was conducted by in a Lightcycler 96 instrument (Roche Diagnostic International). Reactions (20 μL total) were composed of 3.0 μL, 1:25 diluted cDNA, 1 μL of each primer (4 μM), 5 µL UPW and 10 μL Fast SYBR Green Master Mix (Thermo Fisher Scientific, Rehovot, Israel). A standard curve was generated for target and reference genes, assuring R^2^ values of >0.9 gene efficiencies of 2 ± 0.1. All reactions were performed in duplicates under the following conditions: preincubation at 95°C for 1 min followed by 40 cycles of 2-step amplification consisting of 95°C for 10 s and 60°C for 30 s, followed by a melting curve analysis (95°C for 10 s, 65°C for 60 s, and 97°C for 1 s) for ensuring the amplification of a single product. To avoid false positives, a nontemplate control was run for each template and primer pair. Expression levels were calculated using the 2-ΔΔCt method (Livak and Schmittgen, 2001), in which first ΔCt is with regards to the reference gene (β-actin) and the second ΔCt is with regards to the average ΔCt at 17 E. Data are presented as fold change in arbitrary units, relative to 17 E.

### Statistical Analysis

JMP Pro version 15.0 (SAS Institute, Cary, NC) was used for all analyses. Age-related effects were analyzed by ANOVA with significance set at *P* < 0.05.

Mean comparisons were performed by Tukey-Kramer HSD test following for validation equal variances by Bartlett test. Significant differences between means were graphically marked by different letters. Morphometric data (microvilli and villi lengths, microvilli diameters and densities, enterocyte surface areas) are presented as means standard error means from averaged measurements per embryo/chick (n = 3 per timepoint). Gene expression data are presented as means ± standard error means from averaged measurements per embryo/chick (n = 6 per timepoint).

## RESULTS AND DISCUSSION

**Figure 1:**
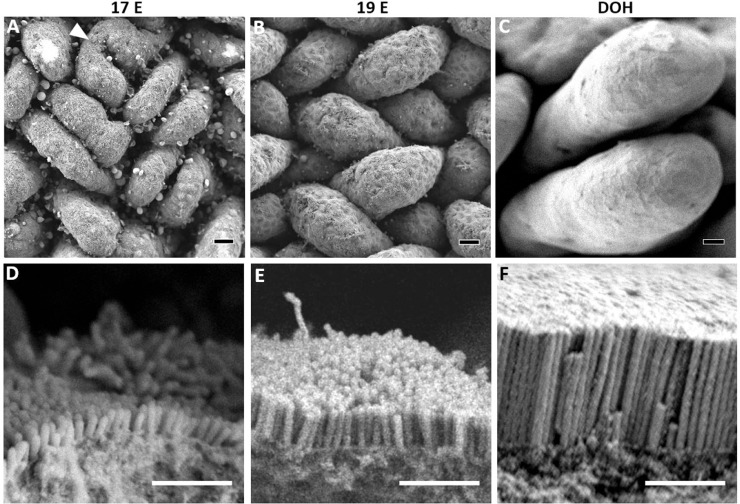
Prehatch brush border maturation. (A–C) Overhead views of prehatch small intestinal villi by scanning electron microscopy. At 17 E (A) villi are rudimentary, and some portray incomplete zig-zag ridge separation (arrowhead). At 19 E (B) villi dimensions increase and at DOH (C) villi become signifincantly longer and finger-shaped. Scale bars, 10 µm. (C–F) Magnified side views of prehatch microvilli at enterocyte apical membranes. At 17 E (C) microvilli are short and loosely-packed. At 19 E (D) microvilli become slightly longer, denser and more uniformly oriented. At DOH (F) microvilli lengths, density and uniformity increase significantly. Scale bars, 1 µm.

Characterizations and measurements of the small intestinal mucosa and brush border morphology during the perihatch period were conducted by scanning electron microscopy (SEM) and light microscopy (**LM**). Prehatch, an overhead view of the small intestinal mucosa at embryonic d 17 (17 E, 4 d before hatch) revealed rudimentary villi structures, some of which portrayed incomplete zig-zag ridge separation (Figure 1A, arrowhead). This is in accordance with previous observations of villi development in chick embryos, in which the initiation of zig-zag ridge separation into individual villi occurred between 16 E and 17 E (Huycke and Tabin, 2018; Shyer et al., 2013). Villi dimensions increased at 19 E (2 d before hatch), with a significant, 57% increase in length in comparison to 17 E (Figure 1B, Figure 3). By DOH, villi assumed dramatically larger, finger-like structuring, and their lengths increased 2.9-fold (*P* < 0.0001) (Figure 1C, Figure 3).

Magnified side-views of trimmed villi revealed that at 17 E, enterocyte apical surfaces were lined with loosely-packed microvilli, measuring less than 0.4 µm in length (Figure 1D, Figure 3). At 19 E, microvilli did not significantly differ in length from 17 E, but portrayed higher uniformity (Figure 1E, Figure 3). By DOH, microvilli were organized in high density and uniform orientation, and their lengths increased 3.1-fold compared to 19 E (*P* = 0.0004) (Figure 1F, Figure 3). These observations indicate that during the last days of incubation, the morphological development of villi (Uni et al., 2003) is accompanied by rapid brush border development, through increasing microvilli lengths, densities, and orientation. These features directly correlate with the progression of enterocyte differentiation and functionality during the last days of incubation (Li et al., 2008; Zwarycz and Wong, 2013; Crawley et al., 2014).

**Figure 2:**
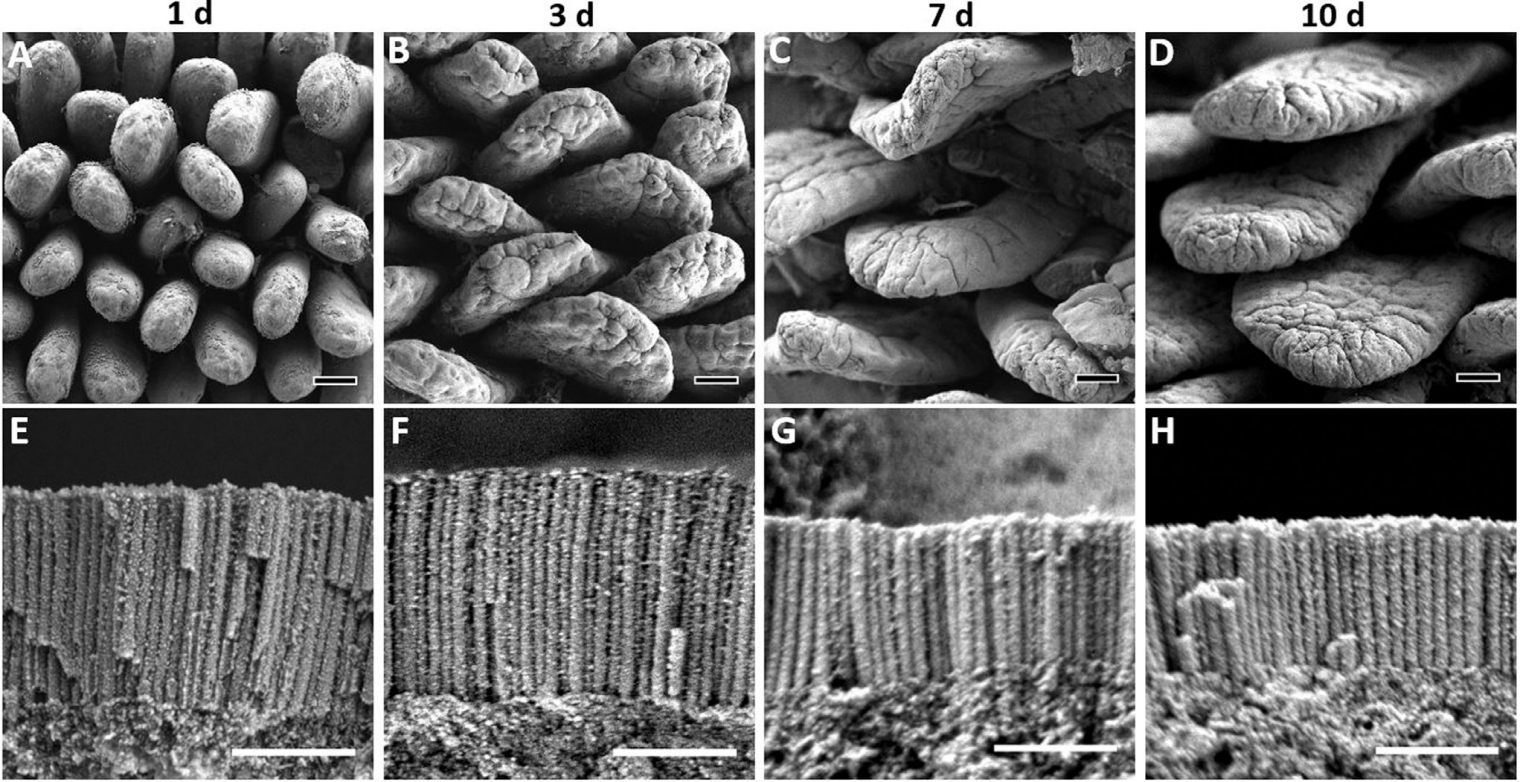
Posthatch brush border maturation. (A–D) Overhead views of posthatch small intestinal villi by scanning electron microscopy. At 1 d (A) villi finger-shaped. At 3 d (B) villi elongate and broaden into tongue-shaped structures. At 7 d (C) and 10 d (D) villi are longer and broader, assuming leaf-shaped structures. Scale bars, 50 µm. (E-H) Magnified side views of prehatch microvilli at enterocyte apical membranes. At 1 d (E) and 3 d (F) microvilli reach maximum lengths. At 7 d (G) and 10 d (H) microvilli lengths decrease. Scale bars, 1 µm.

**Figure 3:**
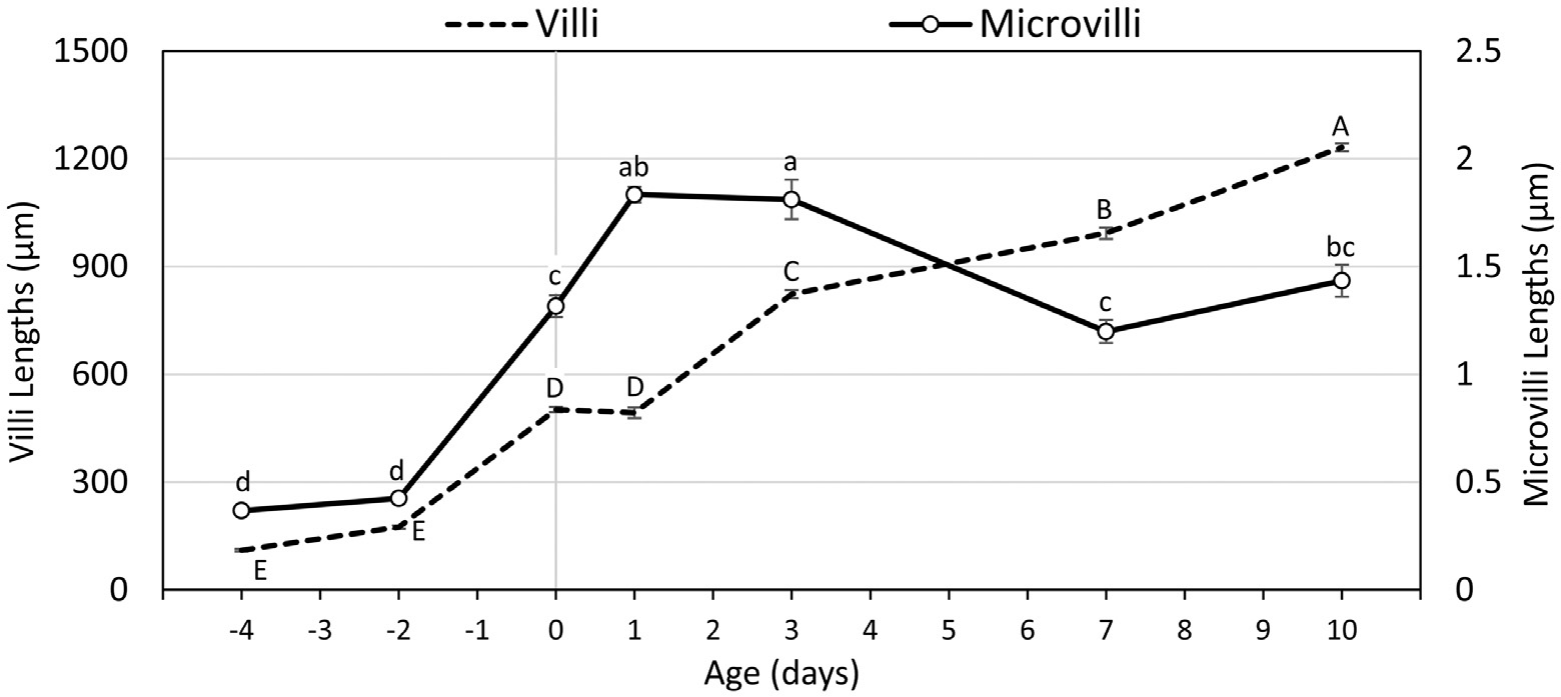
Perihatch villi and microvilli lengths. Villi and microvilli lengths were measured at prehatch ages 17 E (-4), 19 E (-2), day of hatch (0) and post hatch days 1, 3, 7 and 10. Villi lengths (dotted line, left X axis) were measured in 10 villi from each replicate (n = 3 embryos/chicks at each timepoint) by light microscopy (LM). Microvilli lengths (black line, right X axis) were measured in 6 cells from 3 villi from each replicate (n = 3 embryos/chicks at each timepoint) by scanning electron microscopy. Values are means ± standard error means. Significant differences by Tukey-Kramer HSD test are marked by different letters (Uppercase for villi, lowercase for microvilli).

Posthatch, these developmental patterns change, as villi lengths remain stable during the first 24 h (Figure 2A, Figure 3), while microvilli lengths proceed to increase by 40% (*P* = 0.035) (Figure 2E, Figure 3). Between 1 d and 3 d, villi undergo dramatic structural changes, as their bottom portions expand, resulting in tongue-like structuring (Figure 2B), and their lengths increase by 70% (*P* < 0.0001), while microvilli lengths remain stable (Figure 3). At 7 d and 10 d, when the small intestinal mucosa reaches functional maturation (Geyra et al., 2001a; Gilbert et al., 2007; Uni et al., 1998,) villi expand in both lengths and breadths, resulting in broad, leaf-like structures (Figure 2C,D). These villi structures are indistinguishable from 21 d villi (Van Leeuwen et al., 2004), therefore we conclude that villi reach structural maturity around 7 to 10 d. Length measurements at these timepoints reveal surprising pattern changes, as microvilli lengths decrease by 41% between 3 d and 7 d (*P* = 0.001) and did not portray significant changes at 10 d, while villi lengths increase by 20% between 3 d and 7 d (*P* = 0.005) and further increased by 26% at 10 d (*P* < 0.0001; Figure 3).

These results show that microvilli and villi development during the perihatch period are not linear, and follow different patterns. The rate of microvilli elongation exceeds the rate of villi elongation, starting two days prior to hatch and up to 3 d posthatch, while between 3 d and 7 d, villi continue to elongate and microvilli lengths decrease. Previous studies have reported similar increases in microvilli lengths during the final days of embryonic development and first days posthatch (Chambers and Grey 1979; Karcher and Appelgate, 2008), as well as variations in microvilli lengths during the first days posthatch (Mitjans et al., 1997; Karcher and Appelgate, 2008).

**Figure 4:**
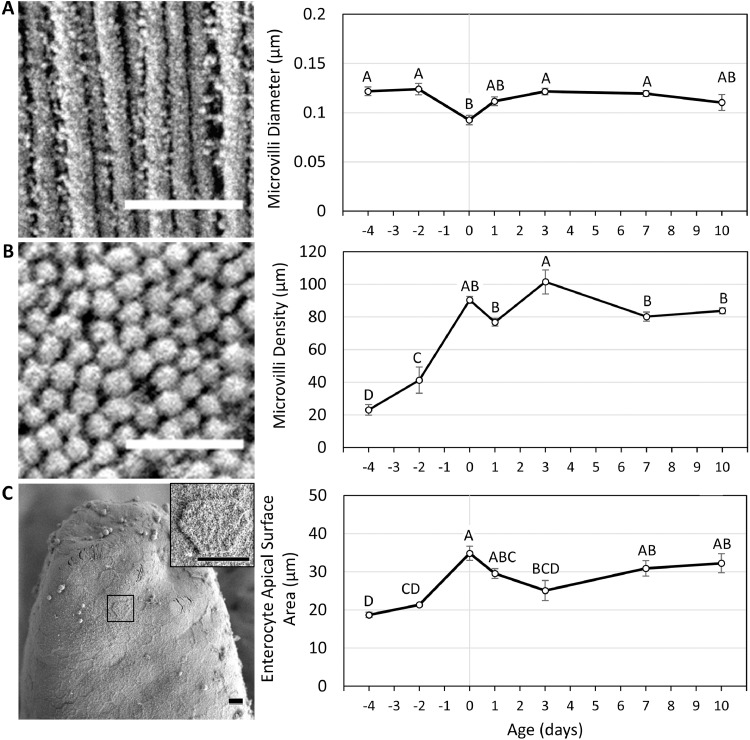
Brush-border morphometric parameters during the perihatch period. Microvilli and enterocyte morphometric parameters were measured at prehatch ages 17 E (-4), 19 E (-2), day of hatch (0) and posthatch d 1, 3, 7, and 10 by scanning electron microscopy (SEM). (A) Microvilli diameters were measured in 6 cells from 3 villi per replicate (n = 3 embryos/chicks at each timepoint). Values are means ± standard error means. Different letters mark significant differences by Tukey-Kramer HSD test. Left panel: representative image from 1 d. Scale bar, 0.5µm. (B) Microvilli densities were measured in 3 to 5 cells from 3 villi per replicate (n = 3 embryos/chicks at each timepoint). Values are means ± standard error means. Different letters mark significant differences by Tukey-Kramer HSD test. Left panel: representative image from 1 d. Scale bar, 0.5 µm. (C) Enterocyte surface areas were measured in 5 individual cells with visible borders from 3 villi per replicate (n = 3 embryos/chicks at each timepoint). Values are means ± standard error means. Different letters mark significant differences by Tukey-Kramer HSD test. Left panel: representative image from 1 d. Scale bars, 5µm.

In order to estimate the extent of surface area expansion by microvilli during the perihatch period, we calculated the microvilli amplification factor (**MAF**) and total enterocyte surface area (**TESA**), as described by Ferrer et al. (1995), by measuring microvilli diameters, microvilli densities and enterocyte surface areas (**ESA**) at each examined timepoint (Figure 4). Our results show that microvilli diameters remained stable throughout the perihatch period, with a single, 25% decrease from 19 E to DOH (*P* = 0.009), followed by a 31% increase up to 3 d (P = 0.016). However, microvilli densities per µm^2^ increased significantly between pre- and posthatch ages, with 77% increase between 17 E and 19 E (*P* = 0.015), and a 2.1-fold increase between 19 E and DOH (*P* < 0.0001), at which microvilli densities measure ≈90 units per µm^2^, similar to intestinal microvilli of adult mice (Pinette et al., 2019) and humans (Helander and Fändriks, 2014). Posthatch, microvilli densities ranged between 77 and 102 units per µm^2^ (Figure 4B).

Enterocyte apical surface areas were measured in perpendicularly oriented cells with prominent borders (Figure 4C). Our results show significant increases between pre- and posthatch ages, with a 63% increase at DOH, compared to 19 E (*P* = 0.002). At posthatch ages, ESAs ranged between 25 µm^2^ and 35 µm^2^ (Figure 4C). The hexagonal-shaped apical surfaces of enterocytes, which were easily identifiable in our high resolution, low magnification SEM images (Figure 4C, inset), allowed for more precise measurements of ESAs in comparison to previous studies, in which enterocyte apical diameters were measured and cell surfaces were assumed to be circular (Ferrer et al., 1995; Karcher and Appelgate, 2008).

**Figure 5:**
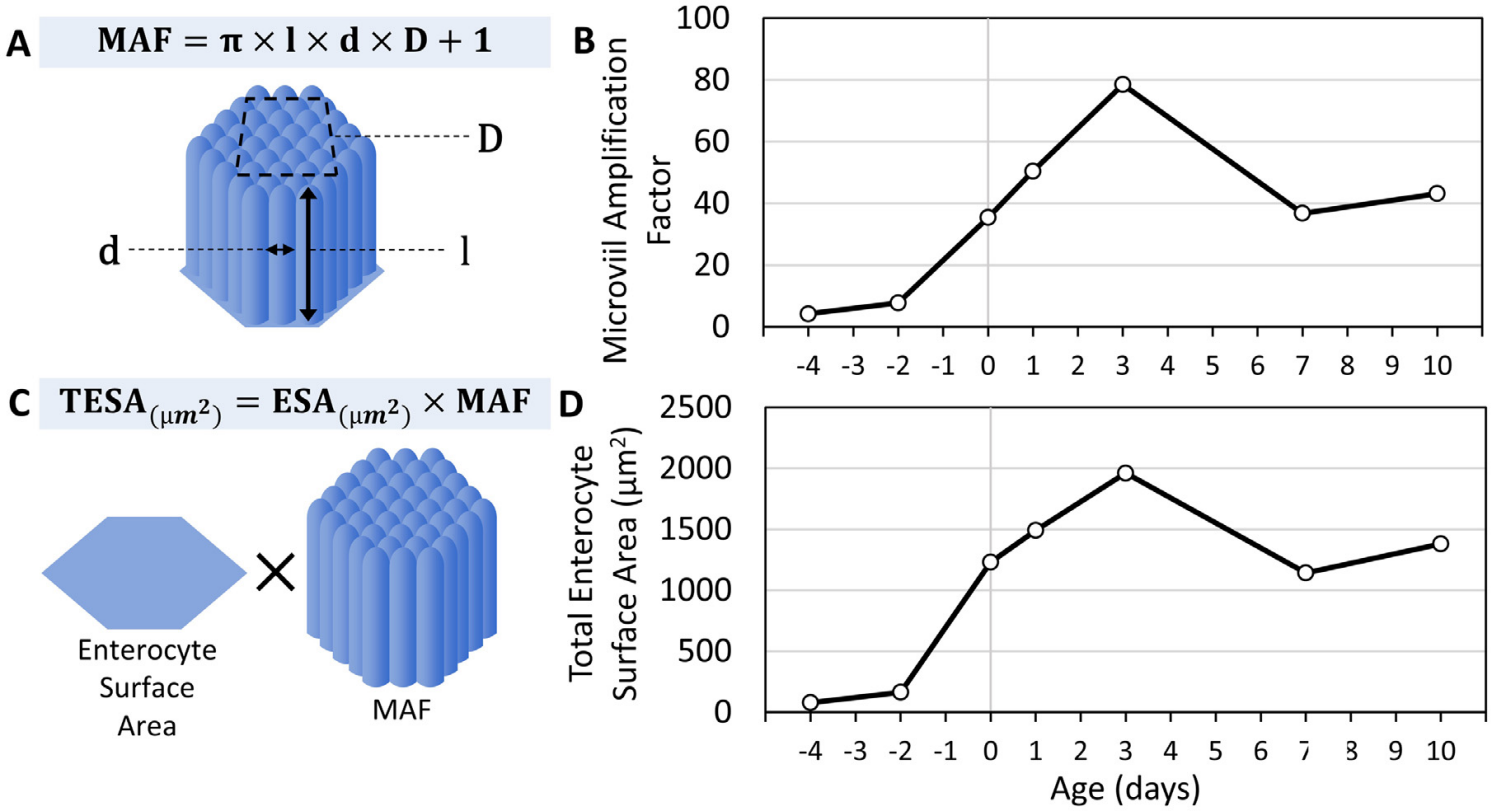
Microvilli amplification factor and total cell surface area during the perihatch period. (A) Microvilli amplification factor (MAF) is calculated by assuming microvilli surface areas as cylindrical and factoring their densities per µm^2^ (Ferrer et al., 1995). (B) Values were calculated using mean microvilli lengths, diameters and densities from Figure 3 and Figure 4. (C) Total enterocyte surface area (TESA) is calculated by multiplying enterocyte surface areas (ESA) by MAF. (D) Values were calculated from mean enterocyte surface areas and MAF values from (B).

Next, data presented in Figure 3 and Figure 4 was calculated for estimating MAF (Figure 5A,B) and TESA (Figure 5C,D). MAF increased from 4.2 and 7.7 at 17 E and 19 E, respectively, to 35.4 at DOH and 50.4 at 1 d. This factor reached a peak value of 78.5 at 3 d, indicating the great capacity of microvilli for enterocyte surface area expansion at this timepoint. By 7 d, MAF decreased to 36.8 and reached a value of 43.2 at 10 d (Figure 5B). TESA followed changes throughout the perihatch period that were similar to those of the MAF, with dramatic increases between 19 E and 3 d, followed by a moderate decrease up to 7 d and stabilization at 10 d (Figure 5D).

In summary, while villi gradually expand and elongate between 19 E and 3 d (Figure 1A–C; Figure 2A,B; Figure 3), MAF and TESA increase nearly 10-fold and 12-fold, respectively. Later on, between 3 d and 10 d, as villi complete their morphological maturation by further expansion and elongation (Figure 2C,D; Figure 3), MAF and TESA decrease and stabilize. These findings demonstrate the great magnitude of enterocyte surface area amplification by microvilli at critical timepoints during the perihatch period, before villi maturation is completed. Since microvilli membranes express nutrient transporters and digestive enzymes, their rapid development during the perihatch period is also critical for intestinal functionality. Accordingly, previous studies reported significantly increased brush border nutrient transporter and digestive enzyme activities, mRNA expression, and protein expression during the final days of embryonic development and first days posthatch (Gilbert et al., 2010; Li et al., 2008; Uni et al., 1998; Uni et al., 2003; Zwarycz and Wong, 2013).

**Figure 6:**
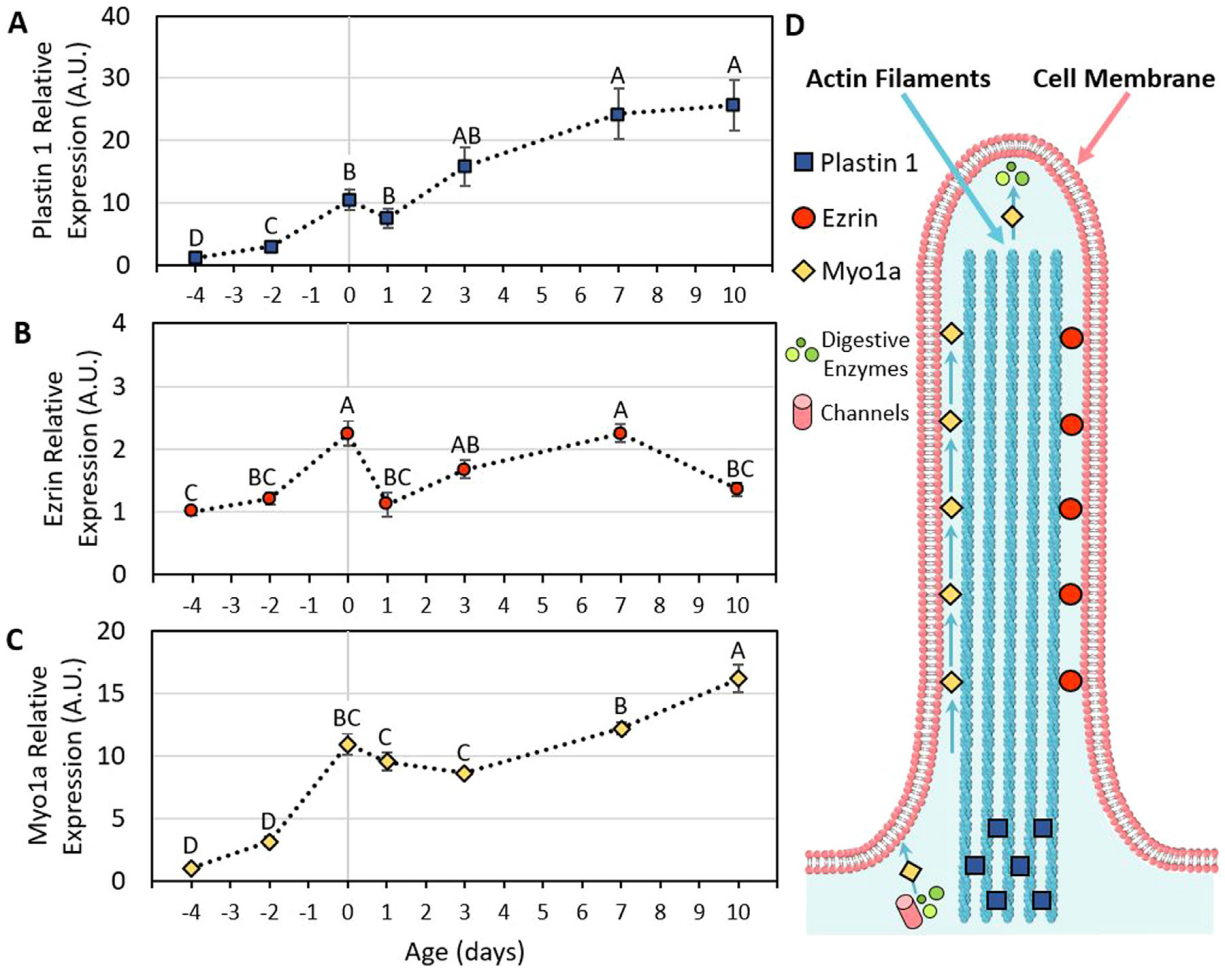
Microvilli structural gene expression during the perihatch period. Relative expression of Plastin 1 (A), Ezrin (B), and Myo1a (C). Values are means from 6 birds at each timepoint ± standard error means. Different letters mark significant differences by Tukey-Kramer HSD test. (D) Graphical representation of Plastin 1, Ezrin, and Myo1a function and localizations within the microvillus. Plastin 1 bundles actin cores and anchors them into the underlying terminal web. Ezrin crosslinks actin bundles to the cell membrane. Myo1a crosslinks actin bundles to the cell membrane and are involved in microvilli motility, nutrient channel and digestive enzyme trafficking to the apical membrane and digestive enzyme secretion though apical vesicle formation (further details in Results and Discussion).

In order to further characterize brush border development during the perihatch period, we evaluated the molecular mechanisms governing microvilli structuring by Real-Time qPCR (Figure 6). First, we examined expression patterns of Plastin 1, an actin bundling protein that is a key regulator in microvilli structuring, length, and terminal web anchoring (Bretscher and Weber, 1980; Ferrary et al., 1999). Between 17 E and DOH, Plastin 1 expression increased 8.7-fold (*P* = 0.005; Figure 6A), in accordance with the observed microvilli elongation and organization patterns (Figure 1D,E). Posthatch, Plastin 1 expression gradually increased, with a 2.3-fold increase in expression at 7 d compared to DOH (*P* = 0.045), and expression plateaued at 10 d (Figure 6A). The maintenance of elevated levels of Plastin 1 expression despite the observed decrease in microvilli lengths after 3 d (Figure 2F,G; Figure 3) indicates the additional roles of Plastin 1 in microvilli maintenance that are not associated with microvilli lengths. Since Plastin 1 regulates microvilli stability through terminal web organization (Grimm-Günter et al., 2009), and the terminal web is responsible for both the vertical alignment of microvilli during their prehatch development and the straitening of apical cell surfaces (Chambers and Grey, 1979), we hypothesize that Plastin 1 is critical for prehatch brush border development as well as posthatch brush border maintenance.

Ezrin, a cross-linker between the cell membrane and microvilli actin cores, is crucial for brush border formation through enterocyte polarization and microvilli structuring (Saotome et al., 2004; Zihni et al., 2014). However, small intestinal Ezrin expression during the perihatch period portrayed only two, 2.3-fold increases from baseline expression at DOH and 7 d, while expression levels did not differ significantly between other pre- and posthatch ages (Figure 6B). This relatively stable expression pattern during the perihatch period can be explained by the fact that structuring and stabilization of microvilli by Ezrin is dependent upon its activation through several enzymes and signaling complexes (reviewed by Crawley et al., 2014). It may therefore be possible that while Ezrin expression levels in the small intestine do not increase significantly between pre- and posthatch ages, its activation state is key for the development and stabilization of microvilli during this period. This hypothesis should be further examined by analyzing Ezrin phosphorylation states throughout the perihatch period, Ezrin activation by PIP_2_ mediated LOK phosphorylation (Pelaseyed et al., 2017), and expression and activity of Ezrin binding proteins and mediators such as EBP50, E3KARP, and NHE3 (Reczek et al., 1997; Yun et al., 1998).

Myosin 1a (Myo1a), the most abundant of intestinal Class I myosins (McConnell et al., 2011) is an actin bundle-cell membrane cross-linker (Mooseker and Tilney, 1975) which is involved in microvilli motility (Meendernik et al., 2019) and is essential for maintaining individual microvilli morphology and brush border organization (Tyska et al., 2005). Myo1a prehatch expression increased 10-fold between 17 E and DOH (*P* < 0.0001; Figure 6C). This dramatic increase in expression may be linked to the role of Myo1a in brush border development, as Meenderink et al (2019) reported prominent Myo1a expression in motile microvilli which drive brush border assembly in differentiating enterocytes. Posthatch expression of Myo1a was stable up to 3 d, increased 1.9-fold between 3 d and 10 d (Figure 6C). This significant increases in expression may be associated not only with microvilli stabilization, but also with increasing intestinal functionality, as Myo1a also contributes cation channel and digestive enzyme trafficking to the brush border membrane (Tyska and Mooseker, 2004; Kravtsov et al., 2012), as well as digestive enzyme-containing vesicle secretion (McConnel et al., 2009). These results provide initial insight into the molecular mechanisms governing intestinal brush border structuring and maintenance in chicken small intestine, during perihatch development.

Taken together, our findings demonstrate rapid, coordinated maturation of microvilli, and their pivotal role in the expansion of the small intestinal surface area during the perihatch period. This process is crucial for sufficient nutrient uptake from feed, while the completion of small intestinal development is underway.

Additional key roles of microvilli in generating and maintaining intestinal functionality include modulation of host-microbiome interactions and protection against pathogens. These functions are achieved by limiting microbe adhesion through negative charging (Bennet et al., 2014), anchoring membrane mucins to form the glycocalyx, a functional microbial-mucosal barrier (Pelaseyed and Hansson, 2020), and secretion of pathogen detoxifying digestive enzymes (Shifrin et al., 2012). Shaping the small intestinal brush-border during early life is crucial for broiler growth and performance, and future studies should focus on factors contributing to microvilli assembly and microbiota-associated interactions during the perihatch period.
